# Exploring role clarity in interorganizational spread and scale-up initiatives: the ‘INSPIRED’ COPD collaborative

**DOI:** 10.1186/s12913-018-3474-2

**Published:** 2018-09-03

**Authors:** Olivia Ly, Shannon L. Sibbald, Jennifer Y. Verma, Graeme M. Rocker

**Affiliations:** 1 University of Western Ontario, London, ON Canada; 2 School of Health Studies, Faculty of Health Sciences, University of Western Ontario, London, ON Canada; 3 The Schulich Interfaculty Program in Public Health, Schulich School of Medicine and Dentistry, University of Western Ontario, London, ON Canada; 4 Department of Family Medicine, Schulich School of Medicine and Dentistry, University of Western Ontario, London, ON Canada; 5 Canadian Foundation for Healthcare Improvement, Ottawa, Canada; 6 Division of Respirology, Nova Scotia Health Authority/Dalhousie University, Halifax, Canada

**Keywords:** Interprofessional collaboration, Professional roles, Organizational factors, Scalability, Chronic disease care, Interorganizational partnership

## Abstract

**Background:**

Role clarification is consistently documented as a challenging process for inter professional healthcare teams, despite being a core tenet of interprofessional collaboration. This paper explores the role clarification process in two previously unexplored contexts: i) in the dissemination phase of a quality improvement (QI) program, and ii) as part of interorganizational partnerships for the care of chronic disease patients.

**Methods:**

A secondary analysis using asynchronous purposive coding was conducted on an innovative pan-Canadian Chronic Obstructive Pulmonary Disease QI program.

**Results:**

Our study reveals that the iterative structure of QI initiatives in the spread phase can offer numerous unique benefits to role clarification, with the potential challenge of time commitment. In addition, the role clarification process within interorganizational partnerships proved to be relatively well-structured, characterized by three phases: relationship conceptualization or early contact, familiarization, and finally, role division. Common strategies in the last stage included the establishment of working groups and new information-sharing networks.

**Conclusion:**

This article characterizes some ways in which providers and organizational partners negotiate their roles in a changing professional environment. As the movement towards integrated care continues, issues of role clarity are assuming increasing importance in healthcare contexts, and understanding role dynamics can provide valuable insight into the optimization of QI initiatives.

## Background

Across the healthcare sector, there is a shift towards models of interprofessional collaboration (IPC) to provide integrated care for patients with complex health needs. Of the Canadian Interprofessional Health Collaborative’s six core competencies for IPC, role clarification is perceived by health professionals to be among the most important for collaboration [1, 2]. Role clarification is the process by which professionals develop a clear understanding of their roles and the roles of others and use this knowledge to achieve patient goals [3]. Role clarity is associated with improved care coordination and professional autonomy balanced with interdependence [4], while ambiguity about responsibilities can lead to conflict and tension [5–7], service duplications or gaps [5, 8, 9], and underutilization of professional expertise [1]. Recognition of role clarification as a valuable collaborative tool is reflected in growing opportunities for students in healthcare fields to develop this skill [10].

The increasing prevalence of chronic disease in North America coupled with patient preference for care in the home presents a growing demand for high quality transitions from hospitals to community services. To ensure the success of new IPC interventions in this field, hospital staff and community practitioners need to be able to understand the diverse roles both within their own teams [11] and within their partner organizations [12]. Current literature describes the processes of role negotiation and clarification within and between hospital teams and reports that open communication [13, 14]; collaborative experiences [15]; change champions [6]; and work environments which value mutual respect, interdisciplinary collaboration, and patient-centred approaches [16] facilitate role clarification. Effective leadership that supports role clarity can range from being ‘manager centred’ to being ‘subordinate centred’ [7] and should plan for the integration of new roles, as well as facilitate and formalize IPC activities [5–7]. Conversely, barriers include poor understanding of others’ scopes of practice [17], limited personal familiarity [15], differences in professional cultures [18, 19], and hierarchical team organization [20].

To our knowledge, literature is lacking on role development both at an organizational level among hospital and community partners in chronic disease transitional services, as well as in spread and scale-up initiatives. The ‘spread and scale up’ strategy is an emerging approach for disseminating change in the healthcare sector, often coupled with quality improvement (QI) initiatives. In contrast to the frequently used ‘scale-up and spread’ framework, in which a successful pilot project is *scaled-up* to benefit more people and then is *spread* across a number of healthcare settings [21], the Canadian Foundation of Healthcare Improvement (CFHI)‘s ‘spread and scale-up’ model recognizes the historical difficulty in spreading innovative practices and focuses exclusively on the dissemination process. The spread phase here continues to refer to the adaptation and implementation of innovations across a geographic area, while the scale-up phase in this case refers to the growth of these secondary programs [22]. Recognizing the complexity of innovation dissemination is the first step in planning for its success. Spread and scale-up QI initiatives could present unique implications for role clarification since they may warrant the participation of a third party that directs different iterations of the program, as well as permit interactions between teams at different sites.

The INSPIRED (Implementing a Novel and Supportive Program of Individualized care for patients and families living with REspiratory Disease) Outreach Program™ is an evidence-based model that addresses gaps in chronic obstructive pulmonary disease (COPD) care by promoting interprofessional collaboration, self-management education, and coordinated outpatient support [23]. Impressive improvements in patient outcomes observed at the Queen Elizabeth II Health Sciences Centre in Halifax prompted the spread phase of implementation, known as the INSPIRED COPD Collaborative. This national dissemination program was launched in September 2014 by the Canadian Foundation for Healthcare Improvement (CFHI) with support from Boehringer Ingelheim (Canada) Ltd., and engaged 19 healthcare teams across Canada. Two prior publications describe experiences of participating in the program and in the spread of the INSPIRED approach [24, 25]. Building on these results and to address existing knowledge gaps, we examined role clarification in the INSPIRED COPD Collaborative to investigate two questions: i) how does the spread of QI initiatives impact role clarification, and ii) how does role clarity develop between hospital and community health organizations working together to transition patients from one system of care to another? Characterizing role clarification in these contexts would further the current understanding of collaborative practice and its implementation.

## Methods

The implementation of the INSPIRED COPD Collaborative occurred between Sept 2014 and Sept 2015. A previous study examining general team processes, health outcomes, context, participant perspectives, partnerships, program reach, and sustainability in the INSPIRED COPD Collaborative was conducted using final reports, surveys, key informant interviews, focus groups, and self-rating exercises [25]. Details about the data collection method can be found in a recent paper [25]. For the current study, notes from key informant interviews (KII) and focus groups (FG), as well as final reports (FR) from the aforementioned study were deemed sufficient to support a contextualized understanding of role development (see Table 1). KIIs, FGs and FRs were gathered at the end of the 12-month period and were designated a number (1–7, 1–3, and 1–19 respectively) for identification in this paper. Because data collection was completed prior to the start of this study, we conducted qualitative secondary data analysis using asynchronous purposive coding methods to identify key themes pertaining to role clarification [26]. Initial coding was conducted by a researcher who was not a part of the original study or the data collection process. Constant discussion throughout data analysis was maintained with a leading researcher of the INSPIRED COPD Collaborative, and all conclusions were verified by both researchers. All research team members supported an iterative and continuous analysis process through discussion and manuscript development.

**Table 1:** Data Sources

Data Source	Total Number	Number of Participants	Number of Teams Represented
Final Reports (FR)	19	N/A	19
Focus Groups (FG)	3	7	5
Key Informant Interviews (KII)	7	31	8

## Results

Below we report our observations on role clarification in the context of a QI initiative in its spread phase and in the context of interorganizational partnerships for chronic disease transition care.

### Role clarification as part of a spread QI initiative

The INSPIRED COPD Collaborative included several unique features that supported role clarification. Firstly, the INSPIRED COPD Change Package came with a number of suggested, loosely defined roles, such as Administrative Lead, Clinical Lead, Project Lead, and Physician Champion. These predetermined roles were helpful in providing teams with an initial structure from which they could further delineate responsibilities according to local resources and needs.

Secondly, one of the most valuable features of the INSPIRED Collaborative was the use of teleconferences, face-to-face workshops, roundtable discussions, and webinars, which allowed teams in various parts of the country to share their experiences. The INSPIRED COPD program encouraged regional adaptation; various approaches to COPD partnerships were being tested simultaneously. Communication among teams consequently permitted the most effective solutions to common problems to surface and to be quickly disseminated. One team’s report, expressing a common sentiment, noted that “access to the INSPIRED staff allowed [them] to learn from people who were walking the same path. Learning from their lived experience [was] most helpful” [FR 16]. Another team said that they “were very impressed with how open all teams were in their experiences and in sharing their resources” [FR 10]. This collective learning process had an important impact on role development. Teams consulted one another on how specific roles, such as Spiritual Care Lead (a non-prescribed role) were carried out, and what salient features made them effective. In particular, many teams struggled with the use of measurement of quality improvement indicators associated with the Measurement and Evaluation Lead role; communication between teams helped to define these responsibilities and provide practical evaluation advice. In addition, strategies that indirectly supported role clarity, such as the clear delineation of care networks, were shared and widely adopted. Several teams did note, however, that differences between regional programs and language barriers could make it difficult to learn from one another, while time commitments, geographical barriers, and financial barriers could interfere with teams’ abilities to attend meetings.

Another consequence of participating in a national, evidence-based, and patient-centred collaborative was a credible profile that helped several teams engage senior leaders. While few teams elaborated further on the subsequent effects on role clarity, one COPD educator stated that the INSPIRED Collaborative “brought COPD to the forefront for [decision-makers] who didn’t know about it but should have” [FG 1], prompting a physician to accompany her on home visits and learn about the importance and challenges of her job. This shared experience led to a greater overall understanding of the role of frontline staff by senior leaders, as well as their inclusion at decision-making tables.

Almost all teams reported that participation in the INSPIRED Collaborative was a demanding time commitment, going above and beyond their current and expected role. Frequent reporting was particularly time-consuming, and many expressed difficulties in attending webinars due to busy and conflicting schedules.

### Role clarification between hospital and community partners in transition care

**Fig. 1:**
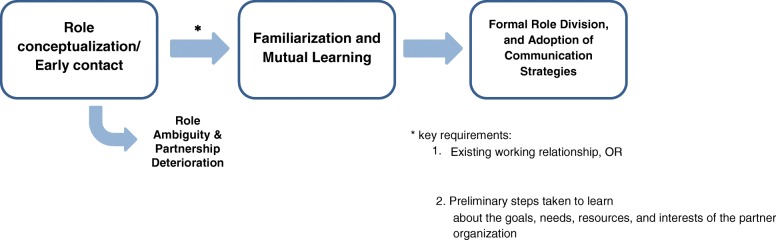
Stages of interorganizational role clarification

The process of role clarification between organizations was characterized by three distinct phases (see Fig. 1). The first phase corresponded to relationship conceptualization or early contact. Hospitals that held existing relationships with organizations in the community noted that “involving [their current partners] into the initiative seemed very logical,” and even the interorganizational relationships of individual practitioners could be leveraged to gain the support of community organizations [KII 3]. Conversely, hospital teams that had not previously collaborated with their community partners expressed uncertainty as to how the different groups would come together. In two teams, presupposed role ambiguity (“[It] wasn’t clear on either side what role they could have” [KII 5]) and lack of awareness of community resources completely stunted further engagement with community resources [FG 1, FR 10]. Nonetheless, some teams with no relevant history of collaboration still succeeded in developing invaluable partnerships. Existing relations thus expedited organizational collaboration but were not necessary for it. However, the lack of any preliminary conception of organizational roles could terminate partnerships before they even began.

To forge successful working relationships, it was crucial for organizations to first learn about one another, particularly for teams without prior partnerships [FR 3, FR 16, KII 4]. Organizations had to understand the goals, resources, and limitations of their partners, as well as the services they wanted to provide, prior to formal role discussions [KII 4]. Knowledge of the diverse individual roles was also valuable, and teams recognized the importance of bringing all the stakeholders to the table both to discuss roles and to foster a shared vision of the initiative. In one exemplar, a hospital hosted over fifty frontline providers in a room at the beginning of the initiative. This event helped providers learn about each other’s roles and the complexity of hospital and community resources. As one participant noted, “you can’t plan where you are going if you don’t know where you are” [FR 16].

Following the opportunity to learn about other organizations and individuals came the task of role distribution and implementation, where again, the collaboration of all stakeholders was essential. Teams that involved a large number of stakeholders tended to subdivide members into representative working groups, with each one responsible for planning specific aspects of the initiative (e.g. self-management support). Through clear assignment of program design tasks to defined groups, working groups supported role clarity on an overarching, project-wide scale. In terms of role clarity on the level of individual professions, strategies to delineate and enforce practitioners’ scopes of practice were diverse. One team conducted exercises that followed the in-patient to out-patient route of care, which helped participants to identify service duplications, to differentiate and standardize the roles of diverse professionals, and to gain a strong understanding of the existing network of care [FR 16]. Others worked to develop an integrated community protocol, such as a regional COPD toolkit [FR 14] and the COPD Pathway project [FR 10], and others simply increased general communication (e.g. phone calls) between professionals [KII 3, KII 2]. Another team introduced a Navigator role to coordinate patient transitions so that the responsibility of understanding the professional landscape fell to one person [FR 9]. Greater overall awareness of the healthcare network prompted almost all teams to establish new patient information transfer strategies. Examples include transfer summaries [FR 16] as well as Transition in Care Reports by respiratory therapists and Visit Reports by out-patient nurses [FR 10]. By promoting the integration of services, such documents also reinforced the roles of different professionals.

Several teams identified the presence of a lead organization or individual to oversee the project, coordinate efforts, maintain accountability, and identify barriers across organizations as a key element for effective collaboration. In addition, increased formal and informal communication allowed stakeholders to learn about their partners and their diverse professional environment over time. Regular communication continued to support a high integration of services even as formal team meetings decreased in frequency. This finding was supported by one team’s past attempts to pursue community collaboration, in which a lack of communication was associated with breaks in acute-to-community care and role ambiguity.

## Discussion

The construction, reinforcement, and defense of professional boundaries are important means by which practitioners understand their profession, establish authority, and maintain their status [19, 20]. Consequently, many individuals experience role changes as threats to their professional identities, particularly when role overlaps require them to relinquish their traditional responsibilities [5, 7] or when professionals have conflicting expectations of each others’ roles [2, 19]. Practitioners can become distrustful and resist collaboration by attempting to reestablish their authority and norms [17, 27], as well as by questioning the value of new interventions [1, 20].

Formal and coordinated interprofessional collaboration is a growing trend and necessary skill in the healthcare sector, emphasizing the integration of health disciplines to provide comprehensive and efficient care for complex health needs. Role clarification is thus a critical process in the shift from traditional systems of silo’d care to an interdisciplinary model, which may require practitioners to adjust their roles to better support collaboration [28, 29]. In this study, we explored the negotiation and delineation of roles in the spread phase of a QI initiative that aimed to foster partnerships between hospitals and community health organizations.

Our data show that QI initiatives can offer unique benefits for role delineation by providing an initial team framework, as well as by providing opportunities for communication between different sites. These meetings, both virtual and in-person, allowed teams to share their experiences, difficulties, and solutions, including those directly or indirectly impacting role clarity. Through its emphasis on multi-tier and interorganizational partnership, the initiative also encouraged in some cases a high degree of role learning between decision makers and frontline staff, while fostering an overall greater appreciation of other health professions along the entire continuum of care in virtually every team. While the process for the former is not clear, teams did note that the national profile of the INSPIRED COPD Collaborative helped to enforce among senior level executives the importance of continuous COPD care and of key health professions. The external accountability of national QI initiatives may thus provide a motive for health care institutions to adopt comprehensive and inclusive improvement strategies. Conversely, the heavy time commitment of participating in the INSPIRED Collaborative reported by all teams may have impeded role clarification. Time constraints are reported as the greatest barrier to resolving role clarity issues as they interfere with the ability of professionals to attend group meetings and to engage in multidirectional, day-to-day consultation [8, 15, 30]. We suggest that future QI initiatives should capitalize on the features that promote role clarification while minimizing the required time commitment.

The rising prevalence of chronic disease highlights the importance of interorganizational partnerships in supporting continuous patient care from the hospital back into their communities and homes. To successfully integrate care, hospital and community partners need to understand what service resources exist and how responsibilities are distributed between organizations and individuals. We found that communication was crucial for role clarification in the INSPIRED interorganizational partnerships, similar to what is reported among smaller, less diverse teams. Communication that is both formal and informal [6, 8, 13], multidirectional, and frequent [14] has been suggested as a prerequisite for role clarification because it allows practitioners to explain their roles, suggest areas of contribution, and ask for clarification on their colleagues’ practice. Furthermore, the importance of regular communication supports existing conceptual propositions that role clarification is an iterative process: roles are not adequately defined once, but are negotiated, adapted, and reinforced through day-to-day activities [6].

In addition, effective leadership that fostered role clarity among organizational teams was uniquely consistent in its high regard for consensus when compared to interprofessional teams within a single hospital or hospital department. Leadership within the latter may use consensus to arrive at decisions or may be seen at the other end of the continuum, in which the team lead or manager assigns roles to individuals with or without their input [6]. Managers in these hierarchical situations may also be solely responsible for orchestrating formal team development opportunities and reconfiguring existing systems to accommodate integrative practices [6]. Conversely, it was found across the INSPIRED COPD Collaborative teams that participation from all stakeholders was highly valued in the program planning process, and that leaders were needed primarily to support coordination and accountability, rather than to assign roles. Role delineation between organizations thus benefitted from a culture of equality.

Finally, the stages and structures for role clarification were more strictly observed at the organizational level than is commonly reported within smaller, less diverse teams. This process in the INSPIRED COPD Collaborative was characterized by role conceptualization and early contact, followed by learning and familiarization, and finally, role division. When hospitals and community services initially came together, the lack of knowledge of what the other could offer could impede further collaboration. Successful teams exceeded this stage by taking time to learn about the other organization’s goals, resources, what they could provide, and what they wanted to provide. This process of familiarization put partners in an informed position to brainstorm which services they wanted to see, from hospital admission to discharge to home visits, and to figure out who could best provide those services. Such consistent phases are not observed in smaller, less diverse, intraorganizational teams, where inclusive planning, mutual buy-in, and consensus are not the only approaches. The organizations in the INSPIRED COPD Collaborative also tended to implemented a number of formalized structures to facilitate interprofessional collaboration, including working groups, action plans upon patient discharge, and new information-sharing systems. Intraorganizational teams in the literature, on the other hand, have employed collaborative strategies that vary in formality. These include, for example, holding occasional meetings to articulate roles [13, 15], participating in interprofessional education interventions, or adopting structural changes, such as new charting practices and team rounds [1]. In acknowledging the high degree of structure in the INSPIRED COPD Collaborative, this study supports a recent publication that describes formalization as a unique characteristic of interorganizational partnerships [31]. We propose that not only does role clarification and formalization influence each other in a reciprocal manner, but also that this need for consistent formal interactions comes from a combination of greater professional diversity, less opportunity for direct contact, and the involvement of more stakeholders.

Role clarity is an essential, but often challenging aspect of IPC that can influence the success or failure of healthcare innovations. Faced with the growing need for efficient systems to deliver high-quality care, an understanding of role clarification processes among both individual professionals and among organizations will greatly improve our ability to design and implement integrative patient-centred programs.

### Limitations

We accept that we have analyzed secondary data from a study that was not originally designed to address issues of role clarity and that this limits the conclusions that we can draw. We acknowledge that organizational culture may impact role clarification among professionals; however, we did not include more information on the topic outside of the introduction in the interest of keeping the paper clear and concise. Furthermore, while the unpublished data revealed that positive organizational culture did contribute to successful teamwork, there was not enough information to link organizational culture to improved role clarity. Another limitation is that this study was conducted within the Canadian healthcare system, which is a unique context given its discrete provincial and territorial divisions. Each division adds a different dimension to care organization and delivery. This could be considered both a strength due to the diversity inherent in the data, but also a limitation due to the potential lack of generalizability to other healthcare systems. Finally, with the diversity of QI programs, our examination of only one collaborative limits the applicability of our findings to other QI programs.

## Conclusions

Role clarification is a dynamic process in the adoption of interprofessional practice. Findings from the INSPIRED COPD Collaborative demonstrate that role clarity develops uniquely in both QI spread initiatives and in interorganizational transition care initiatives. As a QI spread program, the INSPIRED COPD Collaborative supported role clarity by providing a role framework, opportunities for regular communication between teams and stakeholders, and a national profile, although the time commitment posed a challenge for providers. In addition, role clarification in interorganizational partnerships was characterized by more procedural and systematic structure compared to smaller, less diverse healthcare teams. As the trend towards integrated care continues to grow, understanding how providers and organizational partners negotiate their roles in a changing professional arena can provide valuable insight into how to optimize quality improvement initiatives.

## Abbreviations

COPD: Chronic Obstructive Pulmonary Disease
FG: Focus Group
FR: Final Report
INSPIRED: Implementing a Novel and Supportive Program of Individualized care for patients and families living with REspiratory Disease
IPC: Interprofessional Collaboration
KII: Key Informant Interview
QI: Quality Improvement

## References

[1] Suter E, Arndt J, Arthur N, Parboosingh J, Taylor E, Deutschlander S. Role understanding and effective communication as core competencies for collaborative practice. J Interprof Care. 2009;2009 10.1080/13561820802338579. 10.1080/13561820802338579 19142782

[2] Jones ML. Role development and effective practice in specialist and advanced practice roles in acute hospital settings: systematic review and meta-synthesis. J Adv Nurs. 2005; 10.1111/j.1365-2648.2004.03279.x. 10.1111/j.1365-2648.2004.03279.x 15641952

[3] Orchard, C., Bainbridge, L., Bassendowski, S., Casimiro, L., Stevenson K., Wagner, S. J., Weinberg, L., Curran, V., Di Loreto, L., & Sawatzky-Girling, B. A National Interprofessional Competency Framework 2010. http://www.cihc.ca/files/CIHC_IPCompetencies_Feb1210r.pdf. Accessed 15 Nov 2016.

[4] Duner A. Care planning and decision-making in teams in Swedish elderly care: a study of interprofessional collaboration and professional boundaries. J Interprof Care. 2013; 10.3109/13561820.2012.757730. 10.3109/13561820.2012.757730 23343434

[5] Anderson ES, Pollard L, Conroy S, Clague-Baker N. Forming a new clinical team for frail older people: can a group development model help? J Interprof Care. 2014; 10.3109/13561820.2013.853653. 10.3109/13561820.2013.853653 24199595

[6] Brault I, Kilpatrick K, D’Amour D, Contandriopulos D, Chouinard V, Dubois C, Perroux M, Beaulieu M. Role clarification processes for better integration of nurse practitioners into primary healthcare teams: a multiple-case study. Nurs Res Pract. 2014; 10.1155/2014/170514. 10.1155/2014/170514 PMC4322308 25692039

[7] Macintosh M, Goodacre S, Carter A. Organisational influences on the activity of chest pain units during the ESCAPE trial: a case study. Emerg Med J. 2010; 10.1136/emj.2009.073908. 10.1136/emj.2009.073908 20515903

[8] Hepp SL, Suter E, Jackson K, Deutschlander S, Makwarimba E, Jennings J, Birmingham L. Using an interprofessional competency framework to examine collaborative practice. J Interprof Care*.* 2015; 10.3109/13561820.2014.955910. 10.3109/13561820.2014.955910 25208088

[9] Leary M, Schweickert W, Neefe S, Tsypenyuk B, Falk SA, Holena DN. Improving providers’ role definitions to decrease overcrowding and improve in-hospital cardiac arrest response. Am J Crit Care. 2016; 10.4037/ajcc2016195. 10.4037/ajcc2016195 27369032

[10] Hudson CC, Gauvin S, Tabanfar R, Poffenroth AM, Lee JS, O’Riordan AL. Promotion of role clarification in the health care team challenge. J Interprof Care. 2017; 10.1080/13561820.2016.1258393. 10.1080/13561820.2016.1258393 28140704

[11] O’Rouke MW, White A (2011). Professional role clarity and competency in health care staffing – the missing pieces. Nurs Econ.

[12] Amir V, Auslander GK. Inter-organizational collaboration among social workers: the case of community mental health centres and local social service departments in Israel. Br J Soc Work. 2003; 10.1093/bjsw/33.4.557.

[13] Adams TL, Orchard C, Houghton P, Ogrin R. The metamorphosis of a collaborative team: from creation to operation. J Interprof Care. 2014; 10.3109/13561820.2014.891571. 10.3109/13561820.2014.891571 24593331

[14] Gucciardi E, Espin S, Morganti A, Dorado L. Exploring interprofessional collaboration during the integration of diabetes teams into primary care. BMC Fam Pract. 2016; 10.1186/s12875-016-0407-1. 10.1186/s12875-016-0407-1 PMC4736701 26831500

[15] Keefe B, Geron SM, Enguidanos S. Integrating social workers into primary care: physician and nurse perceptions of roles, benefits, and challenges. Soc Work Health Care. 2009; 10.1080/00981380902765592. 10.1080/00981380902765592 19860293

[16] Hilts L, Howard M, Price D, Ridson C, Agarwal G, Childs A. Helping primary care teams emerge through a quality improvement program. Fam Pract. 2013; 10.1093/fampra/cms056. 10.1093/fampra/cms056 22990026

[17] Segar M, Rogers A, Salisbury C, Thomas C. Roles and identities in transition: boundaries of work and inter-professional relationships at the interface between telehealth and primary care. Health Soc Care Community. 2013; 10.1111/hsc.12047. 10.1111/hsc.12047 23656381

[18] Jones R, Bhanbhro SM, Grant R, Hood R. The definition and deployment of differential core professional competencies and characteristics in multiprofessional health and social care teams. Health Soc Care Community. 2013; 10.1111/j.1365-2524.2012.01086.x. 10.1111/j.1365-2524.2012.01086.x 22913320

[19] Liberati EG, Gorli M, Scaratti G. Invisible walls within multidisciplinary teams: disciplinary boundaries and their effects on integrated care. Sci Med. 2016;Soc 10.1016/j.socscimed.2015.12.002. 10.1016/j.socscimed.2015.12.002 26730879

[20] Powell AE, Davies HT. The struggle to improve patient care in the face of professional boundaries. Soc Sci Med. 2012; 10.1016/j.socscimed.2012.03.049. 10.1016/j.socscimed.2012.03.049 22633159

[21] Mittman, B. Factors that influence the scale up and spread of innovations. 2014. https://innovations.ahrq.gov/perspectives/factors-influence-scale-and-spread-innovations. Accessed 5 Jan 2017.

[22] Canadian Foundation for Healthcare Improvement. Spreading the Innovation and Scaling the Innovation. In: Home is where the health is: Scaling up INSPIRED approaches to COPD care*.* N.d. http://www.cfhi-fcass.ca/sf-docs/default-source/documents/inspired-scale/inspired-scale-prospectus-e.pdf?sfvrsn=8. Accessed 24 June 2017.

[23] Rocker GM, Cook D. ‘INSPIRED’ approaches to better care for patients with advanced COPD. Clin Invest Med. 2013; 10.25011/cim.v36i3.19721. 10.25011/cim.v36i3.19721 23739664

[24] Rocker GM, Amar C, Laframboise WL, Burns J, Verma JY. Spreading improvements for advanced COPD care through a Canadian collaborative. Int J Chron Obstruct Pulmon Dis. 2017; 10.2147/COPD.S140043. 10.2147/COPD.S140043 PMC5536231 28794620

[25] Verma JY, Amar C, Sibbald S, Rocker GM. Improving care for advanced COPD through practice change: experiences of participation in a Canadian spread collaborative. Chron Respir Dis. 2017; 10.1177/1479972317712720. 10.1177/1479972317712720 PMC5802658 28612657

[26] Turner BL, Kim H, Anderson DF. Improving coding procedures for purposive text data: researchable questions for qualitative system dynamics modeling. Syst Dyn Rev. 2013; 10.1002/sdr.1506.

[27] Sicotte C, D’Amour D, Moreault M. Interdisciplinary collaboration within Quebec community health care centres. Soc Sci Med. 2002; 10.1016/S0277-9536(01)00232-5. 10.1016/s0277-9536(01)00232-5 12220099

[28] Sweeney P, Kisely S. Barriers to managing mental health in Western Australia. Aust J Rural Health. 2003; 10.1046/j.1440-1584.2003.00504.x. 10.1046/j.1440-1584.2003.00504.x 14641234

[29] Woodhouse G (2009). Exploration of interaction and shared care arrangements of generalist community nurses and external nursing teams in a rural health setting. Aust J Adv Nurs.

[30] Nettings FE, Williams FG. Case manager-physician collaboration: implications for professional identity, roles. and relationships Health Soc Work. 1996; 10.1093/hsw/21.3.216/. 10.1093/hsw/21.3.216 8854126

[31] Karam, M., Brault, I., van Durme, T., & Macq, J. Comparing interprofessional and interorganizational collaboration in healthcare: a systematic review of the qualitative research. Int J Nurs Stud 2018; 10.1016/j.ijnurstu.2017.11.002. 10.1016/j.ijnurstu.2017.11.002 29202313

